# The Segregated Intestinal Flow Model (SFM) for Drug Absorption and Drug Metabolism: Implications on Intestinal and Liver Metabolism and Drug–Drug Interactions

**DOI:** 10.3390/pharmaceutics12040312

**Published:** 2020-04-01

**Authors:** K. Sandy Pang, H. Benson Peng, Keumhan Noh

**Affiliations:** Leslie Dan Faculty of Pharmacy, University of Toronto, Toronto, ON M5S 3M2, Canada; hao.peng@mail.utoronto.ca (H.B.P.); keumhan.noh@utoronto.ca (K.N.)

**Keywords:** segregated flow intestinal model (SFM), traditional model (TM), route-dependent intestinal metabolism, first-pass effect, drug-drug interactions, DDI, in vitro in vivo extrapolations, IVIVE

## Abstract

The properties of the segregated flow model (SFM), which considers split intestinal flow patterns perfusing an active enterocyte region that houses enzymes and transporters (<20% of the total intestinal blood flow) and an inactive serosal region (>80%), were compared to those of the traditional model (TM), wherein 100% of the flow perfuses the non-segregated intestine tissue. The appropriateness of the SFM model is important in terms of drug absorption and intestinal and liver drug metabolism. Model behaviors were examined with respect to intestinally (M1) versus hepatically (M2) formed metabolites and the availabilities in the intestine (F_I_) and liver (F_H_) and the route of drug administration. The %contribution of the intestine to total first-pass metabolism bears a reciprocal relation to that for the liver, since the intestine, a gateway tissue, regulates the flow of substrate to the liver. The SFM predicts the highest and lowest M1 formed with oral (po) and intravenous (iv) dosing, respectively, whereas the extent of M1 formation is similar for the drug administered po or iv according to the TM, and these values sit intermediate those of the SFM. The SFM is significant, as this drug metabolism model explains route-dependent intestinal metabolism, describing a higher extent of intestinal metabolism with po versus the much reduced or absence of intestinal metabolism with iv dosing. A similar pattern exists for drug–drug interactions (DDIs). The inhibitor or inducer exerts its greatest effect on victim drugs when both inhibitor/inducer and drug are given po. With po dosing, more drug or inhibitor/inducer is brought into the intestine for DDIs. The bypass of flow and drug to the enterocyte region of the intestine after intravenous administration adds complications to in vitro–in vivo extrapolations (IVIVE).

## 1. The Intestine–Liver Unit

**Figure 1:**
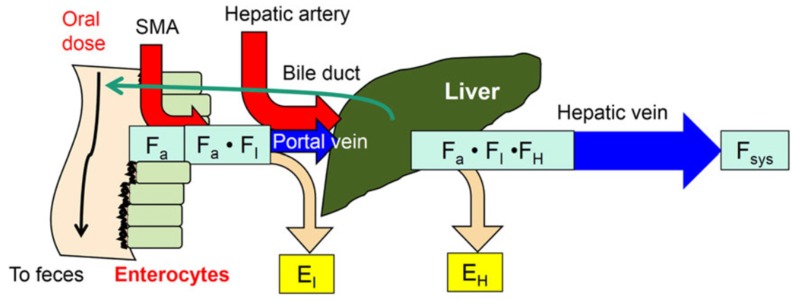
The intestine as a gateway tissue to the liver. Because of intestinal removal [extraction ratio, E_I_ or (1 − F_I_)], the drug entering the liver is reduced, and the liver may further remove the drug with a liver extraction ratio (E_H_) to effect first-pass metabolism. The fraction absorbed, F_a_ and F_I_ or (1 − E_I_), and F_H_ or (1 − E_H_) influence the systemic bioavailability, F_sys_. This figure was reproduced with permission from Noh and Pang [18], Wiley, 2019.

The extent of the absorption of orally administered drugs is controlled by the intestine and liver, which are anatomically linked as a serial unit that is sequentially perfused by the circulation (Figure 1). The intestine is the gateway tissue to the liver and is important for drug absorption and first-pass removal. The superior mesenteric artery (SMA) supplies blood to the small intestine and its venous drainage, together with venous returns from the spleen, pancreas, gallbladder and gastrointestinal tract (GIT) including the stomach, constitute the hepatic portal vein flow (Q_PV_), which is approximately 75% of the total liver blood flow, Q_H_. Together with the hepatic artery (Q_HA_), the remaining 25% of Q_H_, the dual flows collectively perfuse the liver.

The intestine is endowed with absorptive transmembrane transporters in simple columnar, epithelial cells known as enterocytes that line the inner surfaces of the small intestine. These cells contain numerous protrusions known as the villi and microvilli that increase the surface area multiple-fold to absorb drug molecules or nutrients from the gut lumen. Intestinal absorption models have been classically linked to drug properties and the dosage form (pKa, logP, and solubility), as well as the physiology of the gastrointestinal tract (pH, gastrointestinal transit time, gastric emptying time, surface area, and microbiota) that control the fraction of dose absorbed (F_a_) [1,2,3,4,5,6,7,8,9,10,11]. In addition to passive diffusion, absorptive transporters known as the apical solute carrier transporters (SLC), as exemplified by the PEPT1 (oligopeptide transporter 1), OATP1A2, OATP2B1 (the organic anion transporting polypeptide 1A1 and 2B1), MCT1 (the monocarboxylic acid transporter 1), ASBT (apical sodium dependent bile acid transporter) that reclaims bile acids, and OCT (organic cation transporter), facilitate the entry of weak acids and weak bases [12,13,14,15,16,17,18]. Counterbalancing drug entry are the efflux transporters—the P-gp (P-glycoprotein), BCRP (breast cancer resistance protein) and MRP2 (multidrug resistance-associated protein 2) that mediate drug or metabolite secretion back to the intestinal lumen [19,20], and this backward flux tends to reduce the net absorption of solutes. The OSTα and OSTβ (organic solute transporter α and β, half-transporters) transport bile acids out of the enterocytes [21]. It is well recognized that P-gp is capable of secreting highly lipophilic drugs [22,23]. Since lipophilic drugs with high solubility and permeability (Biopharmaceutical Classification System or BCS, Class I) are readily reabsorbed, the excretory function of P-gp is readily nullified [24]. The significance of P-gp, being more abundant distally in the ileum is, therefore, reduced for drugs that are readily reabsorbed [20,23,25,26]. However, for highly soluble but poorly permeable Class III BCS drugs, P-gp is more effective in reducing intestinal drug absorption [7]. It is also notable that drug permeability can be influenced by the pH of the intestinal lumen that becomes more and more basic and in turn, influence the extent of drug absorbed [3,8]. Segment-dependent decline in membrane permeability, reduced surface area from the duodenum to ileum [27] and pH changes along the intestine [8,28] are noted. These variables will modulate the extent of passive drug absorption.

After crossing the intestinal membrane, the drug is met with metabolizing enzymes such as the cytochromes P450 3A (CYP3A) and UDP-glucuronosyltransferases, UGTs [29,30,31,32]. The most abundant CYP isoform is CYP3A4, which exceeds other isoforms such as 2C9, 2C19 > 2J2 > 2D6 that are present in lower quantities [31,33,34,35]. UGT 1A (1A1, 1A6, 1A5, 1A8, and 1A10) and 2B (2B7, 2B15, and 2B17) subfamilies are present to mediate the glucuronidation of morphine, raloxifene, mycophenolate, bisphenol A and gemfibrozil [36,37,38,39,40]. The intestinal metabolic activities for CYP3A4 and some of the UGTs are comparable to, or higher than, those in the liver [31,41,42]. Cytosolic glutathione S-transferases [43,44] are found abundantly, whereas epoxide hydrolases [43] and sulfotransferases (SULT) [45] are present at much lower quantities in the intestine.

The availability of the intestine (F_I_) after intestinal metabolism or secretion is defined as (1 − E_I_) [where E_I_ is the intestinal extraction ratio], and hepatic availability, F_H_, is given by (1 − E_H_) [where E_H_ is the hepatic extraction ratio]. The overall systemic availability, F_sys_, is given by F_a_F_I_F_H_. Following oral (po) drug dosing, the fraction of the dose absorbed (F_a_) is attributed to dosage forms and/or solubility properties, intestinal removal via metabolism or secretion (defined by the intestinal extraction ratio, E_I_), and liver removal (defined as the hepatic extraction ratio, E_H_), respectively. The product of the availabilities, F_a_F_I_F_H_, constitute the net fraction, the systemic availability, F_sys_. For this reason, the intestine and liver are both capable of removing a significant proportion of the orally administered dose, a phenomenon known as the first-pass effect [46]. The extent of intestinal versus liver removal of drugs is therefore intimately related [47,48,49,50]. 

## 2. Reason or Need for Intestinal Flow Models

Although the development of clearance concepts for the intestine has lagged behind that for the liver [51,52,53], there have been some activities trending towards the fabrication of a useful and meaningful intestine clearance model to predict the extent of removal and examine how the intestine influences the rate of liver removal according to the route of drug administration. The correct intestinal model will exert serious implications in terms of drug–drug interactions (DDIs) with inducers or inhibitors, or in terms of in vitro–in vivo extrapolation (IVIVE). 

## 3. Route-Dependent Intestinal Metabolism

Midazolam is a prototypic probe substrate of CYP3A4 metabolism that is often utilized for the screening of CYP3A4 and CYP3A5 activities in inhibition or induction studies [42,54,55,56,57,58]. Midazolam is metabolized by both the intestine and liver [42,59]. For the completely absorbed drug (F_a_ ~ 1), there was a dramatically lower intestinal extraction ratio (E_I_ = 0.08), measured across the arterial and hepatic portal venous blood for midazolam after its intravenous administration among anhepatic patients whose livers were removed during transplantation surgery [59]. In comparison, the mean fraction metabolized across the intestinal mucosa when given intraduodenally was much higher (E_I_ = 0.43). This first, direct evidence uniquely shows route-dependent metabolism of the small intestine. Clinically, the erythromycin breath test relates well to the midazolam unbound liver clearance and not correlated to the intestinal clearance [60]. For radiolabeled (-)morphine that forms morphine 3-glucuronide (M3G) in both the intestine and liver, M3G was absent and undetectable in the vascularly perfused rat intestine preparation when morphine from the reservoir recirculated the rat intestine, a scenario akin to the systemic administration of morphine. This contrasts the copious presence of the radiolabeled M3G metabolite in both the intestinal lumen and reservoir after the intraduodenal administration of morphine into the gut lumen [61]. Additional animal and human studies attest to the same trend of a higher extent of intestinal metabolism after oral (po) than after intravenous (iv) drug administration (Table 1). These examples serve as direct evidence that display route-dependent metabolism of the small intestine. There will be a corresponding route-dependent change in the proportion of liver metabolites formed as well, since the unmetabolized drug leaving the intestine now enters the liver for further processing.

## 4. Intestinal Flow Models: Segregated Flow (SFM), Q_Gut_, and Traditional (TM) Models 

Compartmental models are ill equipped to examine the extent of drug metabolism among metabolizing tissues or organs that are arranged serially. Hence, physiologically based pharmacokinetic (PBPK) modeling of the intestine and liver works a lot better. The approach has been used to appraise the extent of intestine vs. liver removal of drugs [48,49,73,74,75,76,77,78]. Here, the view is that the intestine is perfused 100% by superior mesenteric arterial flow (Q_SMA_), which drains into the portal venous blood (Q_PV_) for the traditional intestinal model (TM), and, upon combining with Q_HA_, these flows in turn perfuse the liver. However, the TM would not explain route-dependent intestinal metabolism on midazolam [59] and morphine [61], which propelled us to develop useful intestinal flow models that can describe this phenomenon. The segregated flow model (SFM) describes a split flow pattern, as proposed by Klippert and Noordhoek [79], with a lower flow rate perfusing the active, enterocyte region (f_Q_ or fraction of the total intestinal flow, <20%) that houses the enzymes and absorptive/efflux transporters, and the remainder flow (>80%) perfusing the non-active, serosal region has since surfaced [80]. With oral administration, the entire dose amount needs to cross into the enterocyte region—the volume of which is conveniently viewed as (f_Q´_V_int_), where V_int_ (or V_I_) is the volume of the total intestine—whereas, for intravenous dosing, <20% of the drug in the circulation reaches the enterocyte region, and this will effectively reduce the rate of drug removal by the intestine. The segregated flow behavior of the intestine is found to explain route-dependent intestinal removal observed for many drugs.

A similar flow model, the Q_Gut_ model [81,82,83], was coined as a minimal model based on the well-stirred model equation for the liver, namely, $F_{I}=\frac{Q_{\mathrm{Gut}}}{Q_{\mathrm{Gut}}{+\mathrm{fu}}_{B}{\mathrm{CL}}_{\mathrm{int}}^{I}}$ [49], after the equation of Yang et al. [83] was corrected upon substitution of fu_B_ for the unbound fraction to intestinal tissue, fu_I_. Since the villous flow (Q_villi_) is 6% of the cardiac output as 19 L/h, the ratio of the Q_villi_/Q_PV_ or f_Q_ value for the Q_Gut_ model is as high as 0.484 for a lipophilic drug such as midazolam [81,82,83]. Notably, f_Q_ is different among these flow models: the SFM (f_Q_ < 0.2), Q_Gut_ model (f_Q_ = 0.484) and TM (f_Q_ =1). The f_Q_ value is expected to affect the extent of intestine and liver removal (E_I_ and E_H_) in the intestine–liver unit with respect to the route of drug administration.

## 5. Equations for Prediction of Route-Dependent Intestinal Removal

**Figure 2:**
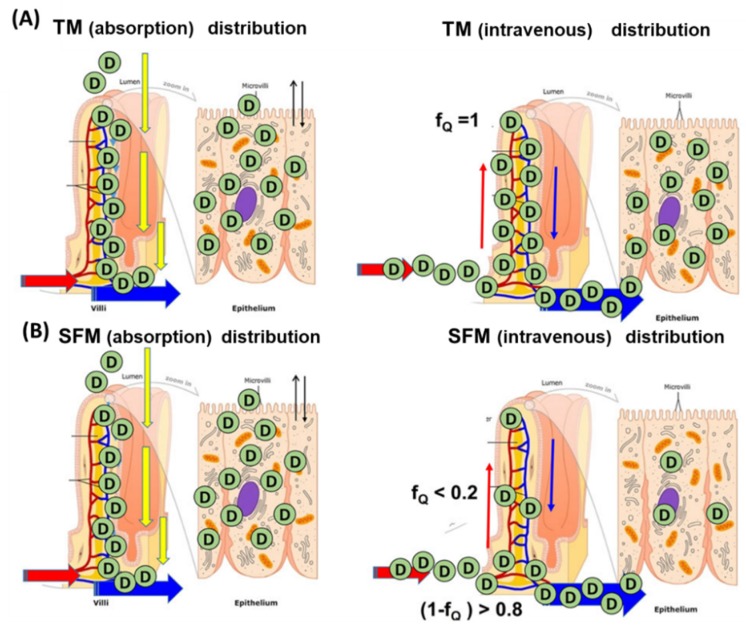
Schematic of drug molecules (D) traversing the intestinal membrane and entering the enterocyte for the tradtional model (TM) (**A**) and segregated flow model (SFM) (**B**). After po admininstration, the drug is absorbed into the enterocyte (yellow arrow) and distributed abundantly in the epithelisum (adjacent) for both the TM and SFM. After intravenous administration, the drug is distributed to the same extent in the epithelium according to the TM (f_Q_ = 1) while the SFM (f_Q_ < 0.2) predicts a much lower distribution of drug in enterocytes. This figure was reproduced with permission from Noh and Pang [18], Wiley, 2019.

There are major differences in drug distribution and therefore intestinal drug clearance when the drug is entering from gut lumen into the villous tip or from the circulation (drug given intravenously) (Figure 2). For the TM, whereby the total intestinal flow perfuses the entire intestine (f_Q_ = 1), there is no difference in the distribution and clearance of drug between oral and intravenous administration when the enterocyte and serosal regions are meshed together (Figure 2A). After po administration, the drug is absorbed into the enterocyte (yellow arrow) and is well distributed in the enterocyte (right graph); the distribution of drug into the enterocyte is also similar after intravenous administration, and the drug is again well-distributed into the enterocyte (f_Q_ = 1). For the SFM (Figure 2B), the extent of distribution after po dosing for a rapidly absorbed drug is similar to that as for TM. Since the enterocyte region is perfused with a lower flow rate (f_Q´_Q_PV_) according to the SFM, its drug extraction ratio for E_I,po,SFM_ is therefore slightly higher than that for the TM, E_I,po,TM_, as the drug is associated with a longer transit time in the tissue [18]. However for iv dosing, there is a reduced distribution of drug reaching the enterocyte due to the reduced intestinal flow (f_Q_ < 0.2), and there will be a smaller intestinal clearance pursuant to intravenous dosing (Figure 2B). Thus E_I,po,SFM_ > E_I,iv,SFM_ or F_I,iv,SFM_ > F_I,po,SFM_ (Figure 2B) when the drug is shunted away from the enterocyte region, especially for highly permeable drugs entering the intestinal tissue from the circulation than from the gut lumen [18,80].

**Figure 3:**
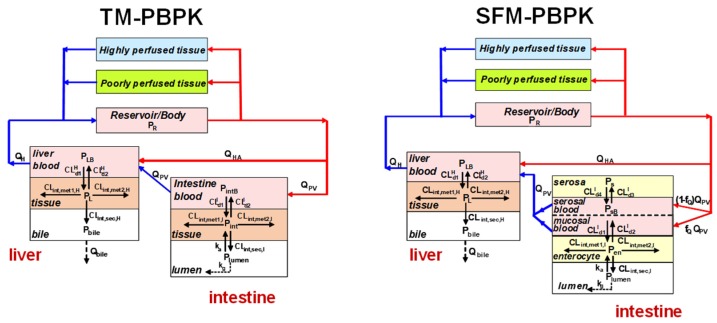
Physiologically based pharmacokinetic (PBPK) models depicting the intestine as a single tissue or compartment for the TM (left) or as the two subcompartments, the enterocyte and serosal subcompartments for the SFM (right), perfused by segregated flows. This figure was reproduced with permission from Sun and Pang [84], Springer, 2010.

The explicit solutions for both the TM and SFM (and Q_Gut_ model) are provided by Sun and Pang [84], who placed the intestine and liver into simple or semi-physiologically based pharmacokinetic (PBPK) models upon viewing both metabolic as well as transport (basolateral influx and efflux) pathways in the intestine and liver (Figure 3). The only difference between the TM and SFM (or Q_Gut_ model) is the presence of an additional intestinal compartment, since the intestine is now denoted as two subcompartments, the enterocyte and serosa, for the SFM and Q_Gut_ model. For simplistic assignment of the volume and flow, f_Q_ x volume or flow are used to designate the enterocyte volume and flow, respectively, and (1 − f_Q_) x volume or flow are used to denote the serosal volume and flow, respectively. A common solution ([Equation (1)] now surfaces to represent the systemic bioavailability with oral administration [84]. This common equation may be used to describe bioavailability, F_sys_, when f_Q_ = 1, 0.484 and <0.2, respectively, for the TM, Q_Gut_ model, and the SFM.

(1) $$\begin{array}{l} \frac{{\mathrm{AUC}}_{\mathrm{po}}{/\mathrm{Dose}}_{\mathrm{po}}}{{\mathrm{AUC}}_{\mathrm{iv}}{/\mathrm{Dose}}_{\mathrm{iv}}}={\;F}_{\mathrm{sys}\;}=F_{a\;}F_{I}F_{H}\\ {\;\;\;F}_{a\;}\left[\frac{f_{Q}Q_{\mathrm{PV}}{\mathrm{CL}}_{d2}^{I}}{f_{Q}Q_{\mathrm{PV}}{\mathrm{CL}}_{d2}^{I}{+(f}_{Q}Q_{\mathrm{PV}}+{\mathrm{fu}}_{B}{\mathrm{CL}}_{d1}^{I}{)[\mathrm{CL}}_{\mathrm{int},\mathrm{met}1}^{I}{+\mathrm{CL}}_{\mathrm{int},\mathrm{met}2}^{I}+{\mathrm{CL}}_{\mathrm{int},\sec}^{I}{(1-F}_{a})]}\right]\left[\frac{Q_{H}^{}{(\mathrm{CL}}_{d2}^{H}{+\mathrm{CL}}_{\mathrm{int},H}^{H})}{Q_{H}^{}{(\mathrm{CL}}_{d2}^{H}{+\mathrm{CL}}_{\mathrm{int},H}^{H}{)+\mathrm{fu}}_{B}{\mathrm{CL}}_{d1}^{H}{\mathrm{CL}}_{\mathrm{int},H}^{H}}\right] \end{array}$$

where ${\mathrm{CL}}_{d1}^{I}$ is the influx transport clearance and ${\mathrm{CL}}_{d2}^{I}$ is the efflux transport clearance. ${\mathrm{CL}}_{\mathrm{int},\mathrm{met}}^{I}$ is the intestinal intrinsic metabolic clearance (for pathways 1 or 2) and ${\mathrm{CL}}_{\mathrm{int},\sec}^{I}$ is the secretory intestinal intrinsic clearance. In the liver, the sum of ${\mathrm{CL}}_{\mathrm{int},\sec}^{H}{\;\mathrm{and}\;\mathrm{CL}}_{\mathrm{int},\mathrm{met}}^{H}{\;\mathrm{is}\;\mathrm{CL}}_{\mathrm{int}}^{H};$ fu_B_ is the unbound fraction in blood, and Q_PV_ and Q_H_ are the portal venous flow and total liver blood flow, respectively. The superscripts I and H denote the intestine and liver, respectively. Notably, the unbound fractions of drug in intestine and liver tissue (fu_I_ and fu_H_) are canceled out in the manipulation.

**Figure 4:**
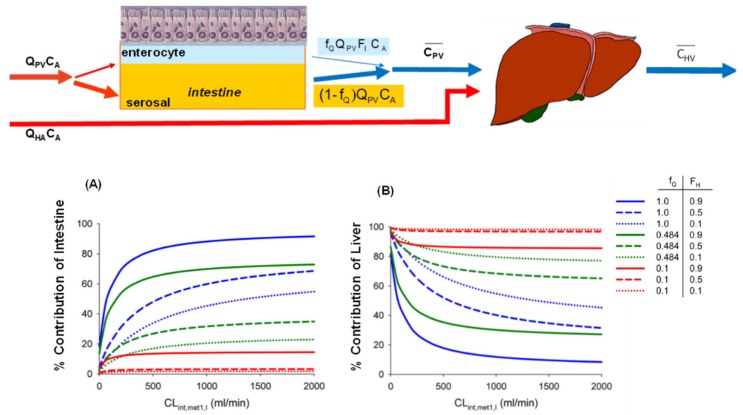
Drug removal by the intestine–liver unit: the intestine controls the substrate flux to the liver. The contributions of intestinal (**A**) and liver (**B**) removal are given by Equations (3) and (4). The drug in the circulation enters two subcompartments of the intestine—the enterocyte and serosal compartments. Removal by the enterocyte but not seroal compartment results in a flow-averaged portal venous concentration,${\bar{C}}_{\mathrm{PV}}$. If intestinal removal is high, the contribution by the liver is opposite and will be low. This figure was modified with permission from Pang and Chow [49], ASPET, 2012.

For a drug in the circulation entering the intestine, the rate of drug removal by the enterocyte is f_Q__ˑ_Q_PV_ (1 − F_I_)·C_A_, but there is no removal by the serosal region (Figure 4). The split flow pattern for the SFM or Q_Gut_ model results in a flow-averaged outflow, portal venous concentration, ${\;\bar{C}}_{\mathrm{PV}}$ [49].

(2) $${\bar{C}}_{\mathrm{PV}}=\frac{f_{Q}Q_{\mathrm{PV}}F_{I}C_{A}{+(1-f}_{Q}{)Q}_{\mathrm{PV}}C_{A}}{Q_{\mathrm{PV}}}=C_{A}[f_{Q}F_{I}+{(1-f}_{Q})]$$

This flow-averaged portal venous concentration is then combined with the arterial concentration (C_A_) to perfuse the liver. Along the same line of reasoning, the rates of removal of drug by the intestine and liver or the fractional contributions are given by,

(3) $$\frac{v_{I}}{v_{I}{+v}_{H}}=\frac{f_{Q}Q_{\mathrm{PV}}{(1-F}_{I})}{f_{Q}Q_{\mathrm{PV}}{(1-F}_{I}{)+E}_{H}\langle Q_{\mathrm{PV}}{[f}_{Q}F_{I}{+(1-f}_{Q}{)]+Q}_{\mathrm{HA}}\rangle }$$

and

(4) $$\frac{v_{H}}{v_{I}{+v}_{H}}=\frac{E_{H}\langle Q_{\mathrm{PV}}{[f}_{Q}F_{I}{+(1-f}_{Q}{)]+Q}_{\mathrm{HA}}\rangle }{f_{Q}Q_{\mathrm{PV}}{(1-F}_{I}{)+E}_{H}\langle Q_{\mathrm{PV}}{[f}_{Q}F_{I}{+(1-f}_{Q}{)]+Q}_{\mathrm{HA}}\rangle }$$

The contributions of the intestine (v_I_) and liver (v_H_) in first-pass removal are hence described by Equations (3) and (4). With f_Q_ values = 1 (left) (TM), = 0.1 (SFM), or = 0.484 (Q_Gut_ model) and with the assumption that Q_PV_ is approximated by Q_SMA_, simulations show that, for a drug entering the intestine from the circulation, the TM predicts the highest intestinal contribution by the intestine–liver unit, whereas the SFM predicts the least; the Q_Gut_ model predicts values somewhere in the middle (Figure 4A). The importance of the intestine increases when the liver possesses a low enzymatic removal capacity (high F_H_). Under the same scenario, results for the %contribution by the liver are the exact opposites, since there is a reciprocal relation to the intestine (Figure 4B). For the SFM, which suggests a lower contribution of metabolism by the intestine for drugs entering from the circulation, the contribution by the liver to first-pass removal is higher than those predicted for the TM and Q_Gut_ model, since there is a greater substrate flux entering the liver that will result in a greater %contribution by the liver, especially for high E_H_ drugs. 

## 6. Is the SFM the Better Intestinal Flow Model Compared to the TM?

Theoretical development of the SFM readily explains the observed higher E_I_ for midazolam and morphine given orally versus intravenously (also Table 1), as do many other drug examples or substrates. When different sets of in vivo or intestinal perfusion data were fitted to the TM versus the SFM, fits to the SFM were all superior over those for the TM. The fitted values of f_Q_ were all <0.2, and the SFM was shown to better the TM statistically among all examples (Table 2). The villous flow pattern to the enterocyte region [85], being a low fraction (<0.2), has also been suggested by Granger et al. [86]. A better discrimination between the TM and SFM occurs when metabolite data are present, as provided by the example of morphine, which forms morphine-3-glucuronide (M3G) by the intestine and liver in the rat in vivo. The discriminatory power for the morphine study was further provided by the biliary versus urinary excretion ratio of the metabolite, M3G, which is unable to cross the liver membrane due to its polarity [87]. The M3G presence in bile suggests that the origin of the metabolite is from the liver. The urinary morphine 3-glucuronide originates from both intestinal and liver metabolism, and the observed ratio of M3G in urine/bile associated with intraduodenal morphine dosing was 2.55-fold that with intravenous morphine administration, as predicted for the SFM [76]. The observations for morphine and morphine 3-glucuronide correlated much better with the predictions from the SFM than from TM.

By contrast, there is practically no difference in the fitted results between the SFM and TM for codeine, the inactive precursor that is *N*-demethylated to form morphine [77]. At first glance, the similarity of both the SFM and TM fits is unique, suggesting that the drug is not subject to intestinal metabolism. For codeine, rat Cyp2d1 (human CYP2D6) is of very low abundance in the intestine, and intestinal metabolism of codeine is very low. For that reason, the agreement of the TM and SFM fits to the codeine data infer a lack of intestine metabolism for codeine. We also recently observed the same pattern for the pan-inhibitor, ketoconazole, after oral and intravenous administration to the rat (unpublished information, Keumhan Noh, Lilly Xu, and K. Sandy Pang).

### 6.1. Implications on Formation of Intestinal and Liver Metabolites

**Figure 5:**
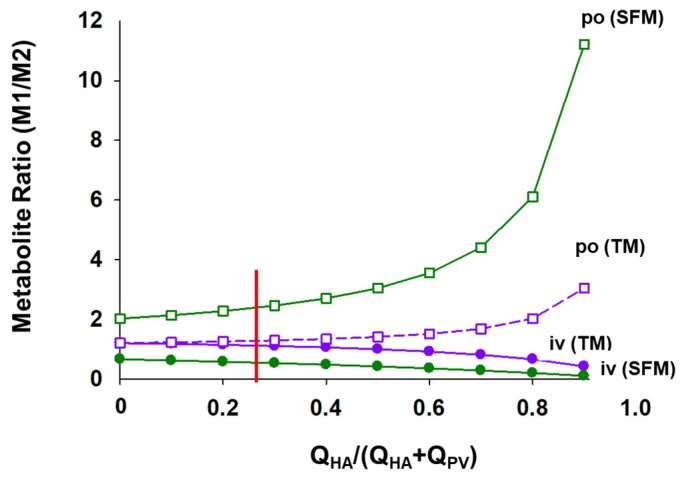
Formation of M1 and M2, specific metabolites formed by the intestine and liver, respecitvely, as simulated by Noh and Pang [18]. The hepatic arterial flow (Q_HA_), normally 25% of total liver blood flow (shown where red line is), delivers the drug directly into the liver, and this contributes M2 formation. Additionally, M2 formation is highest according to the SFM for iv drug administration wherein M1 formation is low due to the low f_Q_. For the TM, the extent of M2 formation is identical for a drug given orally and intravenously, when there is no Q_HA_ flow; the extent increases with increasing Q_HA_. This figure was reproduced with permission from Noh and Pang [18], Wiley, 2019.

Noh and Pang [18] examined the formation of the metabolites: M1 from intestine and M2 from liver, as well as extraction ratios of the intestine with the route of drug administration. For TM, the simulations verified that F_I,po,TM_ = F_I,iv,TM_ for highly permeable drugs, but F_I,po,SFM_ < F_I,iv,SFM_ for SFM and F_I,po,SFM_ < F_I,po,TM_ = F_I,iv,TM_ < F_I,iv,SFM._ The SFM predicts the highest formation of the M1 metabolite with oral dosing but the lowest formation of M1 with intravenous administration; the converse should occur for M2 formation from liver. From M1/M2, the ratio would further unveil that there is more M2 formation arising via the iv route because of direct delivery of drug via the hepatic artery to the liver. Additionally, M1 is less formed according to the SFM for drugs administered iv than po. For this reason, the ratio M1/M2 would always be smaller after intravenous administration according to the SFM as well as TM (Figure 5).

### 6.2. Implications of the SFM on Drug–Drug Interactions (DDIs)

Another reason for properly selecting the intestine flow model is on the prediction of DDI with an inducer or inhibitor. Because >80% intestinal flow bypasses the enterocytes according to the SFM, the route of administration of the inhibitor/inducer, if oral, should be much more effective than the intravenous route, with the underlying reason that the inhibitor/inducer concentrations would be higher in the enterocyte region. Hence, the extent of DDIs is dependent on how the victim drug or inhibitor/inducer is administered and which intestinal flow model, TM or SFM, prevails (Table 3). For midazolam given intravenously (2 mg) or orally (6 mg) to humans, its AUC_iv_ increased 5-fold, whereas AUC_po_ increased 16-fold upon pretreatment with 3 po doses of 200 mg ketoconazole orally at 12 h prior to midazolam dosing, and twice at every 12 h thereafter [57]. For digoxin (1 mg), the inducer rifampin (600 mg daily po for 15 days) produced a dramatic lowering of AUC_po_ but not AUC_iv_ of digoxin due to a 3.5-fold induction of intestinal P-gp protein [20]. In monkeys, ketoconazole inhibited the metabolism of simvastatin, a typical Cyp3a substrate, when given orally and increased the AUC_po_ 5 to 10x, without changing AUC_iv_ for simvastatin given intravenously [90]. For midazolam, oral treatment (50 mg/kg/day for 4 days) of dexamethasone increased the V_max_ values for 1′-hydroxylation and 4-hydroxylation of midazolam in rat intestinal microsomes much more than that with iv dexamethasone [91]. For digoxin given to Wistar rats, purple grape juice (inhibitor of transporter or enzymes) increased the AUC_po_ (73%) but not AUC_iv_ for digoxin [92]. There exist many other examples attesting to this interesting DDI pattern for orally but not intravenously administered victim drugs in the presence of inhibitors or inducers, also given orally (Table 3). These examples confirm the observation that inhibitors or inducers of intestinal enzymes act best after oral administration, since the concentration attained will be highest within the intestine, and the same goes for the victim drug. The inhibition expected for the SFM should be the greatest, and hence this would also create opposite changes in liver metabolism, since inhibition of the intestine leads to a greater flux of substrate towards liver metabolism.

**Figure 6:**
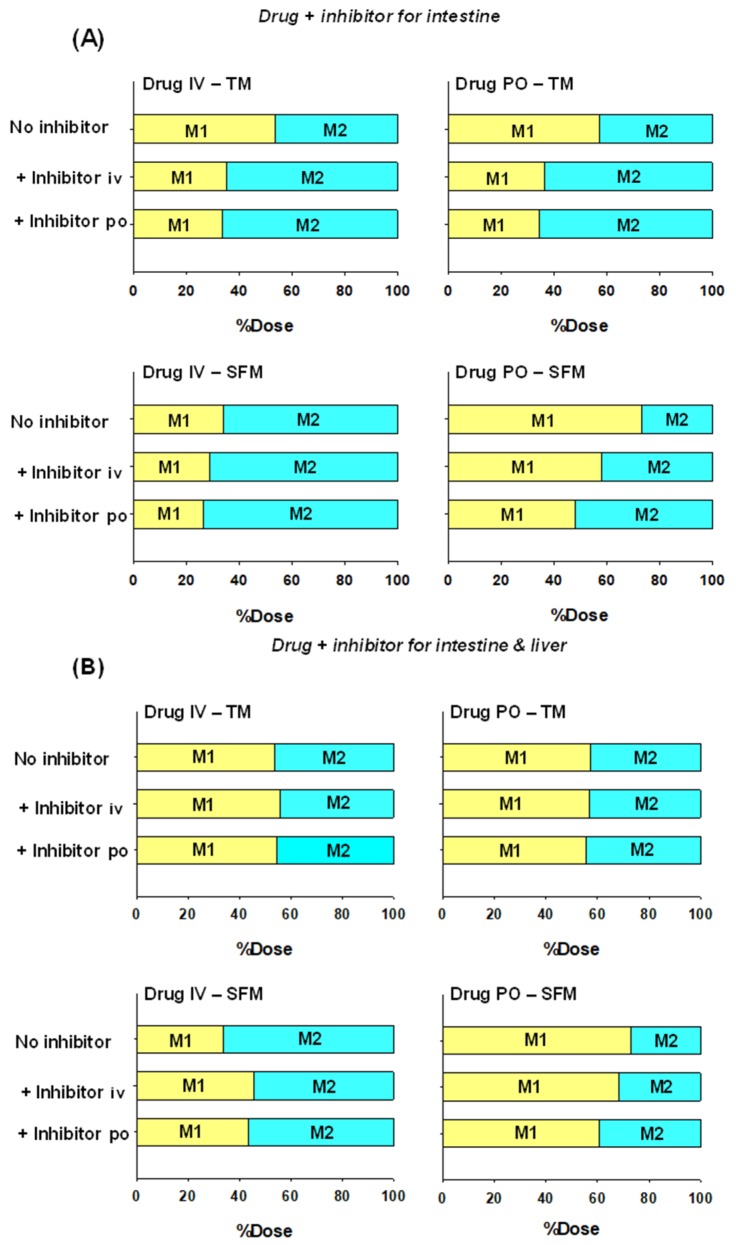
Simulation of intestinally (M1) and hepatically (M2) formed metabolites. For simulation, M1 and M2 were assumed to be inhibited within the intestine only (**A**), and both the intestine and liver (**B**) for a drug example ([18]; data in Table 6 of the reference). The simulation showed that the SFM predicted the highest and lowest M1 formation after oral and intravenous drug admintration, respectively, and the TM predicts a similar extent. The inhibition on intestinal metabolism is the greatest when both the inhibitor and drug are given orally, as predicted by the SFM (**A**). When both intestine and liver metabolism is inhibited, the pattern of change is not readily predictable (**B**). A greater liver inhibition exists after iv drug administration, and the extent of inhibition within the liver can exceed that in the intestine (**B**). This figure was reproduced with permission from data in Table 6 of Noh and Pang [18], Wiley, 2019.

Noh and Pang [18] recently explored the properties of the SFM and TM models with respect to inhibitors via simulations. Within the assigned, limited parameter space set forth for the drug example, the reduction in M1 formation is highest when both inhibitor (intestine inhibition constant, K_i_ = 2 μM) and drug are both given orally, and least or almost unaltered at all when the drug is given intravenously (Figure 6A). Inhibition of metabolism is revealed by the higher drug AUC in the presence of the inhibitor. Often, changes in metabolite patterns are able to reveal inhibition of enzymes within the tissue. For TM, the same extent of M1 formation occurs for both intravenous and oral drug administration, and inhibition of M1 formation is the same after iv or po drug administration. For SFM, a greater extent of inhibition exists for the drug given orally and least when given intravenously. Liver metabolism is in turn affected upon inhibition of the intestinal metabolism, and an inverse relation to that for the intestine is found.

The patterns of intestinal and liver metabolites formed upon inhibition of both the intestine and liver are less revealing as to which tissue is being inhibited, since the proportions of M1 to M2 formed do not always change in the same direction. When inhibition occurring for both the intestine and liver (same K_i_ = 2 μM for M1 and M2 formation), the fluctuations for M1 and M2 are small for the TM and SFM for oral drug administration when inhibition of the intestine is highest. Although inhibition is noted for the victim drug, the extent of M1 formation may even increase due to inhibition of liver metabolism to a greater extent for the drug given intravenously due to the higher input with Q_HA_, with inhibition of the liver being more severe than for the intestine (Figure 6B). It is surmised that the extent of change here depends very much on the parameter space and susceptibility of the intestine versus the liver to the inhibitor and route of administration. But a higher AUC of the drug is strong evidence for the presence of the inhibitor on intestinal and liver metabolism.

### 6.3. Changes in Intestinal and Liver Metabolism with Respect to Flow to Intestine and Liver

Different flow rates to the enterocyte region in the intestine–liver unit would affect intestinal and liver drug processing differentially. An increase in Q_PV_ decreases the E_I,po_ (increased F_I,po_), allowing for more substrate flow to the liver for both the TM and SFM. With the greater substrate flux but faster transit in the liver, the rate of liver metabolism may remain the same although the increase in liver blood flow increases the CL_H_ [47,50]. The converse is also true, with a lower Q_PV_ or Q_SMA,_ an increase in E_I_ and a lower flux to the liver will result.

### 6.4. Implications of the SFM on IVIVE

The IVIVE of transporter function is difficult to deduce when different transit times in GIT, gastric emptying rates, varying pH, and microenvironment exist [116]. The permeability, apical absorptive transporters, and split flow pattern of the intestine to the enterocyte and serosal regions, and efflux transporters complicate the IVIVE picture in the prediction of F_a_ and F_I_. In terms of IVIVE, Kadono et al. [117] employed permeability measurements in artificial membranes to obtain F_a_ from the apparent permeability (P_app_) with the parallel artificial membrane permeability assay (PAMPA) and obtain F_a_ and F_I_ from a scaling factor against a standard such as midazolam using the Yang equation [83]. In addition, IVIVE may be poor for the SFM due to the split flow behavior of the intestinal models, when there is incomplete accessibility of the substrate in circulation to reach enterocytes to fully recruit the intestinal metabolic activity, and this translates to poor IVIVE for the liver. Moreover, methods for identification of intestinal enzymatic activities vary. There are differences in the intestinal functional activity with the mucosal scraping and buffer isolation methods [70,118]. Paine et al. [70] found CYP3A content in each intestinal segment as 30.6, 22.6 and 16.6 pmol/mg mucosal microsomal protein, with similar K_m_ towards midazolam but varying V_max_ values. von Richter et al. [119] showed that the CYP3A4 in isolated enterocytes (76 pmol/mg homogenate protein corresponded to 210 pmol/mg microsomal protein) and was 3.2-fold higher than that in corresponding liver samples, whereas the P-gp content was 7.2-fold higher in enterocyte homogenate than in liver. The CYP3A4 content from the isolated cell method is higher than that from mucosal scraping. Moreover, intestinal metabolism may occur within cells that are shed into the gut lumen that possess copious metabolic activities in the lumen [118]. Nishimuta et al. [120] employed human intestinal and human microsomes to predict the CYP3A intrinsic metabolic clearance for human intestinal microsomes (HIM) versus human liver microsomes (HLM) (CL_int,HIM_ and CL_int,HLM_, corrected by the ratio of CL_int,HIM_ to CL_int,HLM_), and alamethicin-activated HIM for the clearance of UGT substrates. The CYP3A intestinal intrinsic clearance (CL_int,I,CYP3A_) was highly correlated to hepatic intrinsic clearance (CL_int,L,CYP3A_), being 2.2-fold higher in liver, although the correlation was poorer for UGTs. Ito and Houston [34] scaled up the CL_int,H_ with an empirical scaling factor (SF) of 6.2 g protein/kg weight to compensate for the extent of underprediction for IVIVE in rats. Allometric scaling shows that in vitro microsomal data consistently underestimate CL_int,met,I_ and CL_int,met,H_. Hence, scaling and IVIVE remain somewhat empirical approaches.

## 7. Other Intestinal Models 

**Figure 7:**
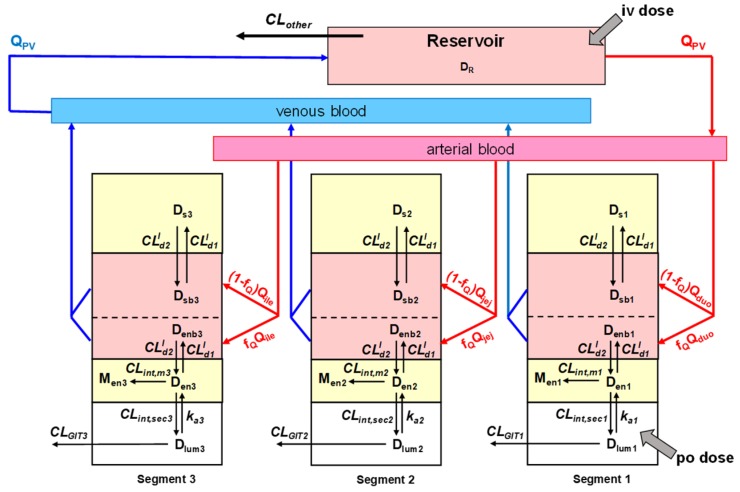
An expanded intestinal flow model—the segmental segregated flow PBPK model depicting the intestine as three different segmental regions with segregated flows to the enterocyte and serosal subcompartments. This figure was reproduced with permission from [121], ASPET, 2003.

**Figure 8:**
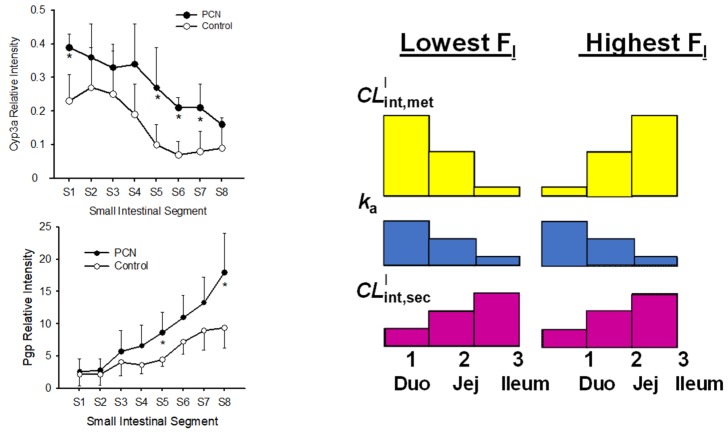
Heterogeneous distribution of Cyp3a and P-gp in the rat intestine, and changes accompanying the inducer, pregnenolone 16α-carbonitrile (PCN) on intestinal bioavailablity. Both P-gp and Cyp3a relative protein expressions were determined by Western blotting (see referecne 26). The scale on the y-axes of the left panel represents an arbitray scale. Segments 1, 2, 7, and 8 are the duodenal, proximal jejunal, distal jejunal and ileal segments, respectively. The symbols, duo and jej of the left panel denote the duodenum and jejunum, respectively. This figure was reproduced with permission from [26], ASPET, 2006.

Our laboratory has extended the SFM to the segmental, segregated flow model (SSFM) to accommodate transporter and enzyme heterogeneity [121]. However, we have oversimplified the segments as a 1/3 of the total volume, flow and permeability characteristics (Figure 7), even knowing that the surface area, permeability, and lengths of the segments of the digestive tract differed [27]. We found higher abundance P450 activity in the proximal region but higher localization of P-gp in the distal region; this pattern produced the lowest availability in drug absorption (Figure 8). This same trend was confirmed by Watanabe et al. [122] years later in a simulation study. The transporter distributions and functions along the intestinal segments reveal similar transporter and drug metabolizing enzyme distribution patterns along the small intestine for rodents and humans (Table 4). Therefore, the rat may be used to predict drug transport across the small intestine in humans. The same extrapolation, however, is not recommended for drug metabolizing enzymes due to the known species differences observed among animal species [123]. The TM- or SFM-PBPK models have been developed to encompass heterogeneity of transporters and enzymes for improved prediction of PK, including polymorphism and sex differences in enzymes, and tease out contributions of intestine and liver in first-pass metabolism (Table 4). Other factors on the physiology of the GIT may also be considered. It is known that the duodenum is the shortest segment and is approximately 1/5 and 1/7 the lengths of the jejunum and ileum, respectively [28]. As shown by the transport of substrates in segments using chamber or single-pass segmental perfusion, drug permeability, revealed with use of a deconvolution-permeability model, is higher in the jejunum [124,125]. Moreover, the pH and transit times in the duodenum, jejunum and ileum differed [28]. Dressman et al. [3] described, in the continuous absorption model, that the GIT is a continuous tube with varying spatial properties on permeability and solubility and pH, surface area, lengths, diameters, gastric emptying [4], highlighting the importance of gastric emptying time, small intestinal transit time, and effective surface area for absorption [5]. There are other models that accommodate variation in villi surface area, in drug permeability along the intestinal segment. Wu [126] applied the SSFM to examine enterohepatic circulation of glucuronides and found that the processes is affected by segmental distribution of enzymes. With accountability of segmental CYP and P-gp activities, reasonable absorption, efflux, and metabolism are observed for midazolam and compound S [25]. 

Commercially available softwares on drug absorption include Simcyp^®^ (advanced dissolution absorption metabolism (ADAM) model is implemented in Simcyp^®^), GastroPlus and GI-Sim [127], and GUT framework [128], which tackle the subject of drug absorption. Although the same input parameters may be used, the software show different F_a_ prediction characteristics depending on the rate-limiting steps of oral drug absorption [127]. The advanced compartmental absorption transit model or ACAT model [9], first conceived by Yu and Amidon [2] as the compartment absorption model [1], has evolved to include permeability (in silico properties derived from chemical structure), logP, pKa, particle size and dose. Dissolution that is based on the Nernst–Brunner modification of the Noyes–Whitney equation is implemented. The influx and efflux transporters [129], pH and pKa, and heterogeneous enzyme distribution are recognized as important processes of the software [2,10,11]. Other considerations include the microbiota and composition. It appears that most of these models deal with dosage form and drug properties and may not have considered the segregated flow behavior of the intestine. A suggestion is for these software developers to consider first finalizing their software based on the absorption of a drug solution while incorporating flow and enzyme/transporter heterogeneity, then combining this to another model with the drug and intestine properties (logP, pKa, particle size, pH, surface area) to consider drug absorption.

## 8. Conclusions

This review has highlighted that metabolite formation and DDIs of the intestine are not well predicted by the traditional intestinal flow model (TM) with respect to the routes of administration of drug and inhibitor. Instead, we recognize the importance of the segregated flow model (SFM) as the premier model to examine intestinal drug metabolism. The evidence in the literature is compelling in support of the SFM based on route-dependent intestinal metabolism. The higher propensity of inhibition with oral and not intravenous dosing is indisputable. Implementation of the SFM is just an additional intestinal compartment away, and this PBPK segregated intestinal flow model (SFM) should be expanded to encompass heterogeneity of transporters and enzymes (SSFM) for improved prediction of PK, including polymorphism and sex differences in enzymes to tease out contributions of intestine and liver in first-pass metabolism. This type of metabolism model could now be coupled with an absorption model to fully investigate the different aspects of F_a_, F_I_ and F_H_. We encourage the use of the more “bottom–up” approach in PBPK modeling to provide mechanistic insight into intestinal metabolism/transport [148] by incorporating the SFM into the model. Another improvement could be made is when the Q_SMA_ is not assumed to equal Q_PV_. The difference in flow (Q_PV_-Q_SMA_) is due to the venous returns from the coeliac and splenic arteries, and stomach and mesenteries. These venous returns would join that from the small intestine (Q_SMA_) and the hepatic arterial flow to perfuse the liver [149,150].

## Figures and Tables

**Table 1:** Examples of route-dependent intestinal metabolism.

Compound	System	Enzyme/Metabolite	Examples	References
Enalapril	Perfused rat intestine–liver preparation	Esterase/enalaprilat	Enalaprilat formed from enalapril after po administration but not systemic administration	[62]
Acetaminophen	Perfused rat small intestine preparation	Ugt1a6/acetaminophen glucuronide	Metabolite observed after intraduodenal but not systemic dosing	[63]
(-)-6-aminocarbovir (6AC)	Perfused rat small intestine preparation	Adenosine deaminase activates (-)-carbovir to 6AC	6AC was highly extracted by intestine after luminal dosing (0.54) compared to reservoir dosing (0.08)	[64]
Morphine	Perfused rat small intestine preparation	Ugt2b1/ morphine 3-glucuronide (M3G)	M3G appeared after intraduodenal but not systemic dosing	[61]
L-754,394, (furanopyridine derivative)	Rats and dogs in vivo and rat liver perfusion	Cyp3a/ epoxide intermediate	Inhibition of L-754,394 and its metabolites by Cyp3a is much greater for po than iv administration of drug	[65]
Cyclosporine	Human in vivo	CYP3A4/AM1 and AM9	Metabolites: AM1 and AM9 are lower after iv compared to po	[66]
Verapamil	Human in vivo	CYP3A4 and 3A5/ norverapamil	Metabolite, norverapamil formation after po > iv	[67]
Hydralazine	Human in vivo	Acetyltransferase/ 3-methyl-striazolo-3,4, α-phthalazine (MTP)	More MTP formation observed after oral dosing than iv dosing	[68]
Cyclobenzaprine	Human in vivo	UGT/ cyclobenzaprine glucuronide (CBG)	Formation of CBG was greater for the oral than for parenteral case	[69]
Midazolam (MDZ)	Human in vivo	CYP3A4/ 1’-OH and 4-OH MDZ	E_I_ after intraduodenal administration >> E_I_ for iv administration	[59,70]
Methyldopa	Human in vivo	SULT/ methyldopa sulfate (MS)	Greater formation of MS after po than iv dosing of M	[71]
Quinidine	Human in vivo	CYP3A/ 3-hydroxyquinidine	More 3-hydroxyquinidine formed via oral compared to iv route	[72]

**Table 2:** Fitted values of f_Q_ in rodents in vivo and in perfusion preparations.

Drug	Fraction of Intestinal Flow to Enterocytes (f_Q_)	Experimental Condition	References
Benzoic acid	0.07	Rat liver perfusion	[88]
Codeine	0.16	Rat in vivo	[77]
Digoxin	0.20	Rat intestinal perfusion	[26]
Digoxin	0.16	Mouse in vivo	[89]
Morphine	0.10	Rat in vivo	[76]
Morphine	0.024	Rat intestinal perfusion	[80]
1,25-Dihydroxyvitamin D_3_	0.11	Mouse in vivo	[87]

**Table 3:** Greater inhibitory or inductive effects after oral administration than iv administration for drug–drug interactions (DDIs) of the intestine.

Compound	Inducer/Inhibitor (Dosing Route)	Enzyme /Transporter	Outcome	Reference
Induction Studies				
Alfentanil	Rifampicin (po)	CYP	Decrease in AUC_po_/AUC_iv_ = 8.2	[93]
Cyclosporin	Rifampicin (po)	CYP	Decrease in AUC_po_/AUC_iv_ = 2.6	[94]
Digoxin	Rifampicin (po)	P-gp	Decrease in AUC_po_/AUC_iv_ = 1.3	[20]
Indinavir	Dexamethasone (po)	CYP and P-gp	Decrease in AUC_po_/AUC_iv_ = 2.3	[24]
Methadone	Rifampicin (po)	CYP	Decrease in AUC_po_/AUC_iv_ = 1.4	[95]
Midazolam	Rifampicin (po)	CYP	Decrease in AUC_po_/AUC_iv_ = 4.4	[96]
Nifedipine	Rifampicin (po)	CYP	Decrease in AUC_po_/AUC_iv_ = 8.5	[97]
Talinolol	Rifampicin (po)	P-gp	Decrease in AUC_po_/AUC_iv_ = 1.7	[98]
Tacrolimus	Rifampicin (po)	CYP	Decrease in AUC_po_/AUC_iv_ = 2.0	[99]
Temsirolimus	Rifampicin (po)	CYP	Decrease in AUC_po_/AUC_iv_ = 1.3	[100]
**Inhibition Studies**				
Alfentanil	Grapefruit juice (po) Troleandomycin (po)	CYP	Increase in AUC_po_/AUC_iv_ = 1.5–2.6	[93]
Atorvastatin	Itraconazole (iv)	CYP and P-gp	AUC_iv +INH_ /AUC_iv_,_control_ = 1.3 AUC_po +INH_ /AUC_po control_ = 2.2	[101]
Cyclosporine	Carvedilol (po) Grapefruit juice (po) Ketoconazole (po)	CYP	Increase of AUC_po_/AUC_iv_ = 1.5–2.8	[66,102,103]
Felodipine	Grapefruit juice (po)	CYP	Increase of AUC_po_/AUC_iv_ = 1.9	[104]
Losartan	Ticlopidine (po)	CYP	Increase of AUC_po_/AUC_iv_ = 1.2	[105]
Midazolam	Clarithromycin (po) Diltiazem (po) Erythromycin (po) Fluconazole (po) Grapefruit juice (po) Itraconazole (po) Ketoconazole (po) Saquinavir (po) Voriconazole (po)	CYP	Increase of AUC_PO_/AUC_IV_ = 1.4–3.2	[57,91,93,106,107,108,109,110,111]
Nifedipine	Grapefruit juice (po) licochalcone A (po)	CYP	Increase in AUC_po_/AUC_iv_ = 1.2–1.4	[112,113]
Saquanvir	Grapefruit juice (po)	CYP	Increase in AUC_po_/AUC_iv_ = 1.7	[114]
Simvastatin	Ketoconazole (po)	CYP	Increase in AUC_po_/AUC_iv_ = 5.0	[90]
Tacrolimus	Ketoconazole (po)	CYP	Increase in AUC_po_/AUC_iv_ = 1.4	[115]

**Table 4:** Heterogeneous distribution of enzymes and transporters in animal and human intestine.

Transporter/Enzyme	Segmental Distribution	References
Animals		
Apical sodium-dependent bile acid transporter (Asbt)	highest at ileum duodenum < jejunum < ileum	[130,131]
Nucleoside transporters (Cnt)	highest in jejunum	[132]
Monocarboxylic acid transporter (Mct1)	duodenum < jejunum > ileum	[88]
Organic cation transporter 1 (Oct1)	duodenum < jejunum < ileum	[133]
Organic cation transporter 3 (Oct3)	duodenum < jejunum < ileum	[133]
Organic anion transporting polypeptide 3 (Oatp3)	highest in jejunum	[133]
Oligopeptide transporter 1 (PepT1)	duodenum > jejunum > ileum	[133]
Multidrug resistance-associated protein 2 (Mrp2)	duodenum > jejunum > ileum	[133]
Multidrug resistance-associated protein 3 (Mrp 3)	duodenum < jejunum < ileum	[133]
Multidrug resistance-associated protein 4 (Mrp 4)	duodenum > ileum > jejunum	[88]
P-glycoprotein (P-gp)	duodenum < jejunum < ileum	[26]
Organic solute transporter α-β (Ostα,β)	duodenum > jejunum > ileum	[21]
Cytochrome P450 3A (Cyp3a)	duodenum ~ jejunum > ileum	[134,135]
Estrone sulfatase	duodenum > jejunum > ileum	[136]
Glutathione S-Transferase (Gst)	duodenum ~ jejunum > ileum	[137]
UDP-Glucuronosyltransferase (Ugt)	duodenum ~ jejunum > ileum	[138]
**Humans**		
ASBT	duodenum < ileum	[139]
OATP2B1	duodenum < ileum	[140]
PEPT1	slightly increasing jejunum > ileum > duodenum duodenum ~ ileum	[19,139]
MCT1	slightly decreasing duodenum > ileum	[19]
CNT11 CNT2	even duo > ileum	[138]
OCT1	even	[19]
OCTN1	duodenum < ileum	[138]
OCTN2	even	[19,139]
MRP3	even	[19]
P-gp	ileum > jejunum > proximal	[19,25,28,140]
BCRP	even jejunum > ileum > duodenum	[19,55,141]
MRP2 mRNA MRP1 protein MRP2 protein	slightly decreasing proximal > distal even	[19,142]
MRP1 to 5 MRP2 to MRP6 MRP4	duodenum < jejunum and ileum	[141,143,144]
CYP3A4	proximal > distal	[25,28]
UGT1A1 UGT1A3 UGT1A4 UGT1A5 UGT1A6 UGT1A7 UGT1A8 UGT1A9 UGT1A10 UGT2B4 UGT2B7 UGT2B10 UGT2B15	duodenum ~ jejunum and ileum duodenum < jejunum and ileum duodenum ~ jejunum and ileum duodenum ~ jejunum and ileum duodenum > jejunum and ileum duodenum ~ jejunum and ileum duodenum ~ jejunum and ileum duodenum ~ jejunum and ileum duodenum and jejunum > ileum duodenum and jejunum < ileum duodenum and jejunum < ileum duodenum and jejunum < ileum duodenum < jejunum and ileum	[145]
SULT1A1, 1A3, 1B1, 1E1 SULT2A1	jejunum < ileum jejunum > ileum	[146]
GST GST	jejunum > ileum jejunum ~ ileum	[147]
